# The Evolution Toward Accreditation

**DOI:** 10.3389/fpubh.2014.00243

**Published:** 2014-11-18

**Authors:** Laura Rasar King, Donna Petersen

**Affiliations:** ^1^Council on Education for Public Health, Silver Spring, MD, USA; ^2^College of Public Health, University of South Florida, Tampa, FL, USA

**Keywords:** accreditation, undergraduate public health education, credentialing, council on education for public health, standalone baccalaureate programs, framing the future task force, critical component elements

The Council on Education for Public Health (CEPH) was established in 1974 to accredit schools and programs of public health, primarily those at the graduate level. For the field, it provides assurance for students, employers, and the public that educational programs meet national standards. For nearly 40 years, the MPH degree has been the recognized entry-level professional degree in the field of public health. However, other public health specialized degrees, particularly those in community health education, environmental health, and health administration have existed at the baccalaureate level for many years. These degrees continue to prepare graduates for certain specialized positions in public health. Accreditation in public health allows educational programs to participate in certain federal funding opportunities, provides them with a marketing advantage to potential students, and provides graduates with opportunities for certain governmental fellowships and jobs.

Over the last decade, undergraduate majors in public health have become increasingly popular. This popularity is seen both at universities with accredited graduate schools or programs in public health, as well as in liberal arts and other types of higher education institutions. A 2008 survey conducted by the Association of American Colleges and Universities, founded in 1915 to represent liberal arts colleges, indicated that 137 of its 837 members, or 16%, offer majors or minors in public health (1). While these numbers represent the most recent official survey data, anecdotal evidence suggests that the number of existing programs today may number as many as 500. These programs have become wildly popular majors for students and attractive to many universities as revenue generators in difficult economic times. Johns Hopkins University has offered an undergraduate major in public health studies since 1976. There were 159 majors in 1998 and 311 majors in 2008. It remains one of the university’s most popular undergraduate majors, currently producing between 110 and 150 graduates per year. At William and Mary, a freshmen seminar on emerging diseases is so popular that it has to be offered in two sections each semester and fills up instantly (1). In 2009, The Chronicle of Higher Education identified public health as one of the five most “up-and-coming majors” likely to be developed at colleges and universities in the coming years (2).

With the growing popularity of and interest in undergraduate public health came an expanded view of what was considered “public health” at colleges and universities around the country. Public health has long been a profession that has benefited from knowledge and expertise contributed by a variety of professions (e.g., medicine, law, business, and social work) and numerous disciplines (e.g., psychology, sociology, and anthropology). Many faculty from these disciplines and professions have applied their expertise to health and health-related questions throughout their careers. On the other hand, few faculty trained in public health find academic homes outside graduate-level public health programs and are unlikely to be found on undergraduate campuses. As such, emerging undergraduate public health majors were of varying foci and tended to reflect existing faculty expertise within the university. Faculty were looking to national organizations, including CEPH, the Association of Schools and Programs in Public Health (ASPPH), and the American Public Health Association (APHA) for guidance on what should be included in the majors they had been asked to develop and seeking a mechanism for quality assurance.

Ongoing conversations among public health academicians revealed a growing unease about the purpose of the undergraduate major in public health. Should students prepared in public health at the undergraduate level be entry-level public health practitioners? Should they be preparing for further professional education in public health or a related profession? In some states, demand is high for entry-level public health professionals trained at the undergraduate level. In certain public health specialty areas, such as community health education, sanitation, and health administration, entry-level practitioners have traditionally been trained at the baccalaureate level. In other areas of the country, and in some employment settings, employers prefer master-level training. Further concern was expressed that the development of undergraduate training in public health would lower the professional bar – that individuals trained at the baccalaureate level would squeeze out MPH graduates in difficult economic times. All these issues converged around the question of whether an accreditation mechanism should be developed for baccalaureate programs in public health.

To determine whether to develop an accreditation system for baccalaureate-level public health programs, the Council, CEPH’s decision-making body, considered two key questions. First, was providing quality assurance for undergraduate public health programs an appropriate “fit” for the organization given that CEPH has accredited only graduate-level programs for almost 40 years? And second, would quality assurance for undergraduate programs in public health be a positive development for the workforce and, ultimately, for the health of the public? The Council determined that the answer to both questions was “yes.”

While the Council had not accredited programs in universities without graduate public health degrees, it had been accrediting schools of public health containing undergraduate degrees for decades and graduate programs that are administratively located with undergraduate degrees since 2007. Graduates from existing programs market themselves as trained in “public health,” but without profession-wide standards or quality assurance mechanisms, the programs vary widely in content, philosophy, and orientation. The Council determined that developing a mechanism to accredit undergraduate public health programs would have a positive impact on the organization’s comprehensive services to the public health profession. The Council also believed that developing a quality assurance program that addresses all levels would have a positive impact on the field of public health by providing a co-ordinating role between degree levels and public and employer expectations of those levels. In short, the Council believed that expansion of its services to undergraduate public health education was appropriate and mission driven.

The Council understood that since there was not yet profession-wide agreement on many aspects of the undergraduate public health major, that development of quality assurance standards around it would need to be a deliberative process. In early 2010, CEPH developed a white paper that outlined the plan for transition to a system of accreditation for undergraduate programs. The paper asked for comments from related organizations about process, content, questions, and potential unintended consequences. Written input was solicited from approximately 30 organizations, representing both public health academia and public health practice, and 11 responded. Comments were strongly supportive for moving forward with exploration of accreditation of public health baccalaureate degrees, but respondents wanted to participate in the design, development, and implementation of any new system.

To ensure a collaborative process, on February 6, 2011, CEPH brought together a group of senior-level public health and educational leaders representing 11 public health and higher education organizations. Members of the group were selected not only for their individual expertise but also to ensure that varying perspectives from government and private public health organizations, potential employers, and academic institutions were represented. The group was asked to provide recommendations to the Council about whether and how to proceed with quality assurance in baccalaureate public health degree programs, particularly those developing without an affiliation with an accredited school or graduate program in public health.

The group arrived at the following consensus statements:

- Given the rapid growth in undergraduate public health in all types of higher education institutions, accreditation might be necessary to assure quality in baccalaureate-level public health majors.
- Accreditation is an iterative, collaborative process that takes time and must involve key stakeholder groups.
- Principles of quality should apply to all baccalaureate-level public health majors, whether in schools of public health, affiliated with graduate public health programs, or in colleges or universities without graduate-level public health training.

The group participated in a brainstorming and prioritization exercise in which they identified core public health content, service learning, and personal and social responsibility among the most important areas of curricular concern. In addition, the group identified important program elements that they believed should be addressed in any potential quality assurance system. These included practitioners as teachers; advising and mentoring students; authentic evaluation of student learning; and recruitment and retention of students, particularly those from underrepresented groups. Further, they agreed that development of criteria for baccalaureate degrees should be informed by ongoing initiatives, such as the Framing the Future Initiative, convened by the ASPPH, to identify curricular components for baccalaureate degree majors.

In 2012, ASPPH convened an expert panel to provide recommendations around undergraduate public health majors as part of its Framing the Future Initiative. The expert panel comprised faculty who worked with undergraduate public health students, practitioners who hired their graduates, and experts on higher education and public health accreditation. Together, this group recommended four critical component elements of an undergraduate public health education that included background domains, public health domains, cumulative experience and field exposure, and cross-cutting areas (3). While the report of the expert panel expanded on each of these areas, it also identified nine public health content domains for undergraduate public health majors. These content areas included an overview of public health; the role and importance of data in public health; identifying and addressing population health challenges; underlying science of human health and disease; determinants of health; project implementation; overview of the health system; health policy, law, ethics, and economics; and health communication. This expert panel report was essential to moving undergraduate public health education from something defined at the university level alone, to something defined by a profession.

From the expert panel report, CEPH was able to draft the curricular criteria for what it called Standalone Baccalaureate Programs (SBP). These undergraduate programs are those not affiliated with a graduate school or program in public health. In early 2013, CEPH convened two focus groups composed of designated leaders of public health majors at a diverse array of universities. The focus group participants gave feedback on the draft criteria and multiple changes were made. Following a period of open public comment on the criteria, the Council adopted them in October 2013 (the full criteria for accreditation are available on the CEPH website at http://ceph.org/assets/SBP-Criteria.pdf). In February 2014, CEPH accepted its first baccalaureate applicants for accreditation.

The accreditation process for SBP is nearly identical to that of schools and graduate programs in public health. The process includes the preparation of a detailed self-study document by the program followed by an on-site visit by a team of peer reviewers who interview faculty and administrators, students, alumni, and community members. Following the visit, the site visit team provides a detailed written report outlining compliance with each accreditation criterion. The Council reviews all the available information and makes a decision about accreditation. The process takes approximately 2.5–3 years from initial application to final accreditation decision. Following initial accreditation, programs are monitored annually and the full process is repeated in 5 years – subsequent re-accreditation processes occur every 7 years. Preparing a program to undertake the accreditation process can take time; thus, the number of applicant and accredited baccalaureate programs expected over the next few years is modest, but will expand over time. Working together with the early applicant programs and the field, CEPH expects to refine its processes and criteria as lessons are learned and promising practices are shared. New information and resources will be updated regularly on the CEPH website at http://ceph.org/constituents/programs-baccalaureate-level/.

At the time of this writing, just a few months after accepting the first applications, CEPH has 12 SBP applicants for accreditation. The list of applicants can be located on the CEPH website at http://ceph.org/accredited/applicants/. The applicants are diverse in many ways, including, program size, geographic location, and delivery model. One-third of them are located in a university with a separately administered graduate program or school of public health. While most note that they are preparing graduates for entry-level public health jobs in a variety of sectors, the aim of others is to provide pre-professional education for graduates to enter health professional schools like medicine, nursing, or pharmacy or non-health professional schools, such as law or urban planning. Nobody is certain whether graduates from a public health major will provide a pipeline to graduate public health education, to another health professional school, or to any number of other paths they may take. One thing that is certain, no matter what these graduates do over the next 5 years or over the next 30 years of a long career, they will have an intellectual foundation that will allow them to understand how their chosen field impacts and is impacted by health. They will understand health determinants and disparities. This popular undergraduate major will bring us one step closer to health in all policies and health in all professions.

## Conflict of Interest Statement

The authors declare that the research was conducted in the absence of any commercial or financial relationships that could be construed as a potential conflict of interest.
